# DFusion: Denoised TSDF Fusion of Multiple Depth Maps with Sensor Pose Noises

**DOI:** 10.3390/s22041631

**Published:** 2022-02-19

**Authors:** Zhaofeng Niu, Yuichiro Fujimoto, Masayuki Kanbara, Taishi Sawabe, Hirokazu Kato

**Affiliations:** Nara Institute of Science and Technology (NAIST), Ikoma 630-0192, Nara, Japan; yfujimoto@is.naist.jp (Y.F.); kanbara@is.naist.jp (M.K.); t.sawabe@is.naist.jp (T.S.); kato@is.naist.jp (H.K.)

**Keywords:** depth fusion, TSDF, sensor noises

## Abstract

The truncated signed distance function (TSDF) fusion is one of the key operations in the 3D reconstruction process. However, existing TSDF fusion methods usually suffer from the inevitable sensor noises. In this paper, we propose a new TSDF fusion network, named DFusion, to minimize the influences from the two most common sensor noises, i.e., depth noises and pose noises. To the best of our knowledge, this is the first depth fusion for resolving both depth noises and pose noises. DFusion consists of a fusion module, which fuses depth maps together and generates a TSDF volume, as well as the following denoising module, which takes the TSDF volume as the input and removes both depth noises and pose noises. To utilize the 3D structural information of the TSDF volume, 3D convolutional layers are used in the encoder and decoder parts of the denoising module. In addition, a specially-designed loss function is adopted to improve the fusion performance in object and surface regions. The experiments are conducted on a synthetic dataset as well as a real-scene dataset. The results prove that our method outperforms existing methods.

## 1. Introduction

Depth fusion is of great importance for many applications, such as augmented reality applications and autonomous driving. Many methods have been proposed in this area and truncated signed distance function (TSDF) [1] is one of the most famous. However, TSDF requires manual adjustment on its parameters, possibly leading to thick artifacts. To address this problem, some depth fusion methods have emerged with improved performance. Methods such as [2,3] use surfel-based or probabilistic approaches to generate 3D representations, which may be a voxel grid, a mesh or a point cloud. In addition, compared with these classical methods, convolutional neural network (CNN) based methods have shown advantages in the fusion performance. However, their results still suffer from noisy input, which results in missing surface details and incomplete geometry [4].

**Figure 1:**
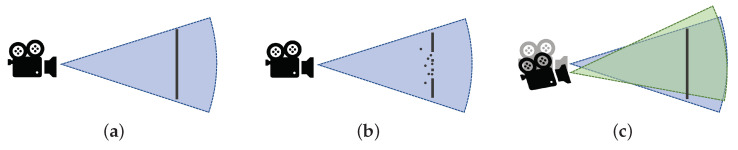
Illustration of the sensor noises. (**a**) Sensor without noises. (**b**) Depth noises. (**c**) Sensor pose noises.

The data acquired by depth cameras inevitably contain a significant amount of noise. Although researchers have proposed many methods to remove the noise, most of the works only focus on removing the noise caused by depth maps but neglect the noise of camera poses (pose noises for simplicity). Figure 1 illustrates the two types of noises. Figure 1a shows the situation where there is no noise and a plane is in the sight of the camera. If there are depth noises, the noise may be outliers or missing data, as shown in Figure 1b, which leads to noisy TSDF volumes. As for the pose noise, Figure 1c provides an example when the camera has translation and rotation error compared with Figure 1a, which causes troubles when integrating the TSDF updates due to the inaccurate extrinsic data. Both types of noises may have adverse impacts on depth fusion results. However, there are only a few works that focus on removing noises for TSDF fusion, even given the fact that both types of noises are inevitable.

**Figure 2:**
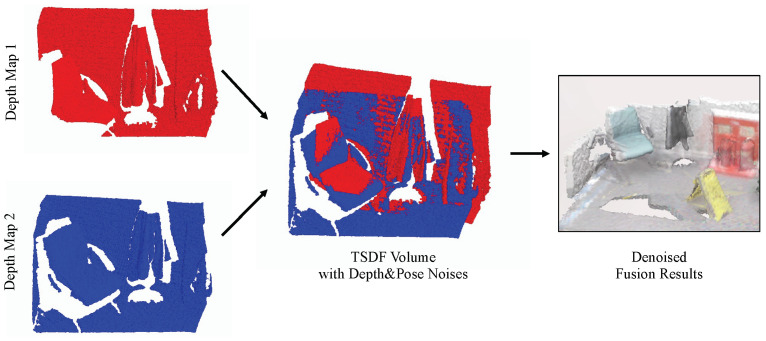
DFusion can minimize the influence of both types of noises.

RoutedFusion method [4], as an example, considers the depth noise and aims to obtain a robust TSDF volume against different levels of depth noise. It uses depth maps derived from synthetic datasets and puts random noises into the depth maps. However, in the fusion process, the camera pose they use is the ground-truth pose from the synthetic dataset, so that the results can only be robust against depth noise, but not against pose noise. In this paper, we propose a method named DFusion that considers not only depth noises but also pose noises, as shown in Figure 2. To the best of our knowledge, this is one of the earliest research that tries to avoid the performance drop caused by pose noises.

Generally, depth fusion is conducted with 2D convolutional models. However, when considering the pose noise, it is better to remove the noise with the 3D representation because it is challenging to recognize and remove the surface shifts in the 2D space. Therefore, we firstly adopt a Fusion Module, as the first part of DFusion, with the same setting as the fusion network in the RoutedFusion method, to fuse the depth maps with camera poses into a TSDF volume. After gaining the integrated TSDF volume, we design a Denoising Module, an UNet-like neural network, as the second part of DFusion to denoise the TSDF volume. Since the input of the Denoising Module is a 3D volume, 3D convolutional layers are utilized to obtain the 3D features. Skip connections are used to avoid the vanishing gradient problem, which is prone to occur due to the small value of TSDF volume.

For training the networks, we utilize a synthetic dataset which can provide the ground-truth value of depth maps and camera poses. The model is trained in a supervised manner. In addition to the commonly-used fusion loss, several specially-designed loss functions are proposed, including a $L_{1}$ loss for all voxels in the whole scene and $L_{1}$ losses over the objects and surfaces for better fusion performance on these regions.

In sum, the contributions of this work are as follows:

- We propose a new fusion network named DFusion, which considers both depth noises and pose noises in the fusion process. DFusion can avoid the performance drops caused by both types of noises, and conduct accurate and robust depth fusion.
- We design new fusion loss functions that focus on all the voxels while emphasizing the object and surface regions, which can improve the overall performance.
- The experiments are conducted on a synthetic dataset as well as a real scene dataset, measuring the actual noise levels with the real-world setting and demonstrating the denoising effects of the proposed method. The ablation study proves the effectiveness of the proposed loss function.

## 2. Related Works

### 2.1. Depth Fusion and Reconstruction

#### 2.1.1. Classical Methods

TSDF fusion method [1] is one of the most important classical fusion methods that fuses depth maps with camera intrinsics and the corresponding viewpoints, i.e., camera poses, into a discretized signed distance function and weight function, thereby obtaining a volumetric representation. It has been adopted as the fundamental in the majority of depth map fusion based 3D reconstruction, including KinectFusion [5], BundleFusion [6], and voxel hashing [7,8]. However, the depth maps always involve noises but all these methods update a wider band to deal with the noise, as a result, there are noise artifacts, especially outlier blobs and thickening surfaces, on the results.

In contrast to the voxel-based method, there are some reconstruction approaches that update the results in different ways. For example, Zienkiewicz et al. [9] introduce a scalable method that fuses depth maps into a multi-resolution mesh instead of a voxel grid. Keller et al. [10] design a flat point-based representation method [2], which utilizes the input from the depth sensor directly without converting representations, thereby saving the memory and increasing the speed. In addition, the surfel-based approach that approximates the surface with local points is adopted for reconstruction [2,11]. The unstructured neighborhood relationship can be built by this approach, although it usually tends to miss connectivity information among surfels. MRSMap [12], as an example, integrates depth maps into a multi-resolution surfel map for objects and indoor scenes.

Some researchers also regard the depth map fusion process as a probabilistic density problem [3,12,13,14], considering various ray directions. Yong et al. [15] estimate the probability density function based on the original point cloud instead of the depth map and use a mathematical expectation method to decrease the complexity of computation. In [16], the marginal distribution of each voxel’s occupancy and appearance is calculated by a Markov random field along with the camera rays. However, all these classical methods have limitations to balance reconstruction quality, scene assumptions, speed and spatial scale due to the large and complex computation but limited memory.

#### 2.1.2. Learning-Based Methods

Along with the development of deep learning methods, there exist lots of proposals that fuse and improve the performance of classical 3D reconstruction [17]. For example, ScanComplete [18] method completes and refines the 3D scan with a CNN model, which can deal with the large-scale input and obtain the high-resolution output. RayNet [19], which combines a CNN model with Markov random fields method, considers both local information and global information of the multi-view images. It can cope with large surfaces and solve the occlusion problem. Based on Mask R-CNN method [20], Mesh R-CNN [21] detects objects in an image, then builds meshes with a mesh prediction model and refines the meshes with a mesh refinement model.

Specifically, in many learning-based approaches, TSDF fusion is still one of the important steps [22]. OctNetFusion [23] fuses the depth maps with TSDF fusion and subsequently utilizes a 3D CNN model to deal with the occluded regions and refines the surfaces. Leroy et al. [24] propose a deep learning-based method to achieve multi-view photoconsistency, which focuses on matching features among viewpoints for obtaining the depth information. Similarly, the depth maps are finally fused by TSDF fusion. RoutedFusion [4] also fuses the depth maps based on the standard TSDF fusion. Different from other methods, it reproduces TSDF fusion by a CNN model, which predicts the parameters of volume and weight, then the volumetric representation can be updated with new volume and weight sequentially.

Compared with the classical method, deep learning-based methods show advantages in handling thickening artifacts and increasing diversity and efficiency. In addition, existing methods pay little attention to the noise problem during the fusion process. Our method adopts a part of RoutedFusion models to fuse the 3D volume firstly, then combines a special-designed neural network to remove the noise, thereby improving the performance of the depth fusion.

### 2.2. Denoising/Noise Reduction

Most of the works consider the noise as the depth noise and try to remove the noise at the beginning of the fusion process. The authors in [3,25] adopt Gaussian noise to mimic the real depth noise derived from the depth sensors, then achieve the scene reconstruction. Cherabier et al. [26] also remove some regions of random shapes, such as circles and triangles, to simulate the missing data. In RoutedFusion [4], the authors add random noise to the depth maps and propose a routing network that can remove the random noise, then use a fusion network to fuse the denoised depth maps into a TSDF volume. The experiments prove that the routing network has a significant effect on improving accuracy.

Another way to cope with the noise is to refine the 3D representation directly. NPD [27] trains the network by utilizing a reference plane from the noiseless point cloud as well as the normal vector of each point while PointCleanNet [28] removes the outlier firstly then denoises the remaining points by estimating normal vectors. Han et al. [29] propose a local 3D network to refine the patch-level surface but it needs to obtain the global structure from the depth images firstly, which is inconvenient and time-consuming. Zollhöfer et al. [25] propose a method that utilizes the details, such as shading cues, of the color image to refine the fused TSDF volume since the color image typically has a higher resolution. A 3D-CFCN model [30], which is a cascaded fully convolutional network, combines the feature of low-resolution input TSDF volume and high-resolution input TSDF volume to remove the noise and refine the surface. However, all these methods only consider either the outliers of the 3D representation or the noises caused by depth maps. In our method, we design a denoising network with 3D convolutional layers, which remove the noise for the TSDF volume without any other additional information. In addition, we take the noise of both depth maps and camera poses into account; thus, the network is robust against not only depth noises but also pose noises.

## 3. Methodology

### 3.1. TSDF Fusion

Standard TSDF fusion, which is proposed by Curless and Levoy [1], integrates a depth map $D_{i}$ with the camera pose and camera intrinsic into a signed distance function $V_{i}\in R^{X\times Y\times Z}$ and weight function $W_{i}\in R^{X\times Y\times Z}$. For location *x*, the integration process can be expressed as follows:

(1) $$\begin{array}{c} V_{i}\left(x\right)=\frac{W_{i-1}\left(x\right)V_{i-1}\left(x\right)+w_{i}\left(x\right)v_{i}\left(x\right)}{W_{i-1}\left(x\right)+w_{i}\left(x\right)} \end{array}$$

(2) $$\begin{array}{c} W_{i}\left(x\right)=W_{i-1}\left(x\right)+w_{i}\left(x\right) \end{array}$$

It is an incremental process, and $V_{0}$ and $W_{0}$ are initially set as zero volumes. In each time step *i*, the signed distance $v_{i}$ and its weight $w_{i}$ are estimated according to the depth map of the current ray, then are integrated into a cumulative signed distance function $V_{i}\left(x\right)$ and a cumulative weight $W_{i}\left(x\right)$.

However, in the traditional way, the parameters are tuned manually, so that it is a heavy task and difficult to exclude artifacts and maintain high performance. In RoutedFusion [4], the TSDF fusion process has been conducted in a convolutional network, named depth fusion network, which is trained to tune the parameters automatically. The input of the fusion network is depth maps, camera intrinsics and camera poses. The depth map is fused into the previous TSDF volume with the camera intrinsic and camera pose incrementally. The main purpose of RoutedFusion method is to deal with the noise of the TSDF volume caused by the noise on depth maps. To remove the depth noise, the authors firstly adopt the depth maps with random noises for training, then use a routing network to denoise the depth maps before fusing them with the fusion network.

In a real application, however, the pose noise is also inevitable. Therefore, in our method, the inputs include noised depth maps and noised camera poses.

### 3.2. Network Architecture

The proposed DFusion method mainly includes two parts: a Fusion Module for fusing depth maps and a Denoising Module for removing the depth noises and pose noises. These two modules are trained independently, with different loss functions.

**Figure 3:**
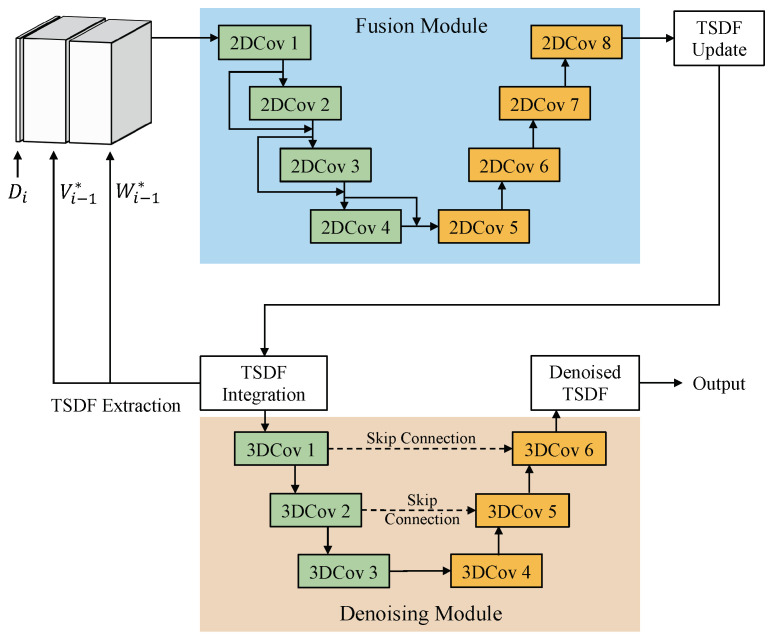
The DFusion model.

**Fusion Module.** The Fusion Module follows the design of the fusion network proposed in the RoutedFusion method [4]. It fuses depth maps incrementally with a learned TSDF updating function, using the information of camera intrinsics and camera poses. Then the TSDF update will be integrated to form a TSDF volume for the whole scene. The process of the Fusion Module is illustrated in the upper part of Figure 3. Although RoutedFusion can remove the depth noise, its denoising process is implemented as a pre-processing network, i.e., the routing network as metioned in Section 3.1, rather than the Fusion Module which is used in our method. Also, different from the RoutedFusion method, we consider not only the depth noise but also the pose noise, the latter of which is much more obvious when fusion is finished than before/during fusion. Therefore, we add a post-processing module to deal with both of these two types of noises.

**Denoising Module.** After obtaining the TSDF volume, the Denoising Module is designed to remove the noise of the TSDF volume. The input of the Denoising Module, which is also the output of the Fusion Module, is a TSDF volume with depth noises and pose noises. Since it deals with a 3D volume, we adopt 3D convolutional layers instead of 2D convolutional layers, aiming to capture more 3D features to remove the noise (as using 3D convolutional layers is a natural choice for tasks such as 3D reconstruction [30] and recognizing 3D shifts are extremely difficult for 2D convolutions). As shown in Figure 3, the Denoising Module is implemented as an UNet-like network, which downsamples the features in the encoder part and upsamples them back to the original size in the decoder part. Skip connections are added among encoder layers and decoder layers.

In the training phase, to mimic the noises of real-world applications, we add random noises to the ground-truth depth maps and camera poses of the dataset. Therefore, the output of the Fusion Module, as well as the input of the Denoising Module, is noisy and needs to be fixed. For the depth noise, we add the noises $B_{d}$ that follow a normal distribution to all pixels *P* in the depth maps (following the solutions in [4,23]). This process can be represented as

(3) $$P^{\prime }:=P+B_{d},$$

and

(4) $$B_{d}∼N\left[0,\sigma_{d}\right],$$

where $\sigma_{d}$ is the pre-defined scale parameter. This parameter should be set to reflect the actual noise levels of the applications. We set $\sigma_{d}=0.005$ following [4,23].

As for pose noises, we add the noise to translation matrix *T* and rotation matrix *R*, respectively. Firstly, given a random translation error $B_{t}$, a random rotation error $B_{r}$, two random unit vectors $n_{t}=\left(n_{1},n_{2},n_{3}\right)$ and $n_{r}=\left(n_{4},n_{5},n_{6}\right)$ (respectively, for translation and rotation errors), the noised translation matrix and rotation matrix are calculated as follows.

(5) $$\begin{array}{cc} T^{\prime } & :=T+n_{t}\cdot B_{t}\\ R^{\prime } & :=R+\mathrm{Rodri}(n_{r},B_{r}), \end{array}$$

where $\mathrm{Rodri}(n_{r},B_{r})$ follows Rodrigues’s rotation formula and it can be represented as:

(6) $$\left(\begin{array}{ccc} n_{4}^{2}\left(1-cosB_{r}\right)+cosB_{r} & n_{4}n_{5}\left(1-cosB_{r}\right)-n_{6}sinB_{r} & n_{4}n_{6}\left(1-cosB_{r}\right)+n_{5}sinB_{r}\\ n_{4}n_{5}\left(1-cosB_{r}\right)+n_{6}sinB_{r} & n_{5}^{2}\left(1-cosB_{r}\right)+cosB_{r} & n_{5}n_{6}\left(1-cosB_{r}\right)-n_{4}sinB_{r}\\ n_{4}n_{6}\left(1-cosB_{r}\right)-n_{5}sinB_{r} & n_{5}n_{6}\left(1-cosB_{r}\right)+n_{4}sinB_{r} & n_{6}^{2}\left(1-cosB_{r}\right)+cosB_{r} \end{array}\right)$$

In addition, $B_{t}$ and $B_{r}$ also follow the normal distribution.

(7) $$\begin{array}{c} B_{t}∼N\left[\mu_{t},\sigma_{t}\right]\\ B_{r}∼N\left[\mu_{r},\sigma_{r}\right] \end{array}$$

Since there is no existing method that adds artificial pose noises to improve the denoising performance, the value of μ and σ is decided based on a real scene dataset. More details are given in Section 4.2.

### 3.3. Loss Functions

Since there are two modules in the network, i.e., Fusion module and Denoising module, the total loss function involves two parts as follows.

**Fusion Loss.** The loss function of the Fusion Module is expressed as follows:

(8) $$\begin{array}{c} L_{F}=\sum_{a}\lambda_{1}^{F}L_{1}\left(V_{local,a},V_{local,a}^{\prime }\right)+\lambda_{2}^{F}L_{C}\left(V_{local,a},V_{local,a}^{\prime }\right), \end{array}$$

where $V_{local}$ and $V_{local}^{\prime }$ are two local volumes along ray *a*, respectively, from the the network output and from the ground-truth. $L_{1}$ is the L1 loss and can be represented as

(9) $$\begin{array}{c} L_{1}\left(V,V^{\prime }\right)=\frac{\sum_{v_{m}\in V,v_{m}^{\prime }\in V^{\prime }}|v_{m}-v_{m}^{\prime }|}{\left|V\right|} \end{array}$$

In addition, we use the cosine distance loss $L_{C}$ (on the signs of the output volume and ground-truth volume) to ensure the fusion accuracy of the surface, following the setting in [4], which can be represented as

(10) $$\begin{array}{c} L_{C}\left(V,V^{\prime }\right)=1-cos\left(sign\left(V\right),sign\left(V^{\prime }\right)\right), \end{array}$$

where sign() is to get the signs of the inputs and cos() is to get the cosine values of the angles between the input vectors.

In addition, $\lambda_{1}^{F}$ and $\lambda_{2}^{F}$ are the weigths for the loss terms and are emperically decided as 1 and 0.1 [4], respectively.

**Figure 4:**
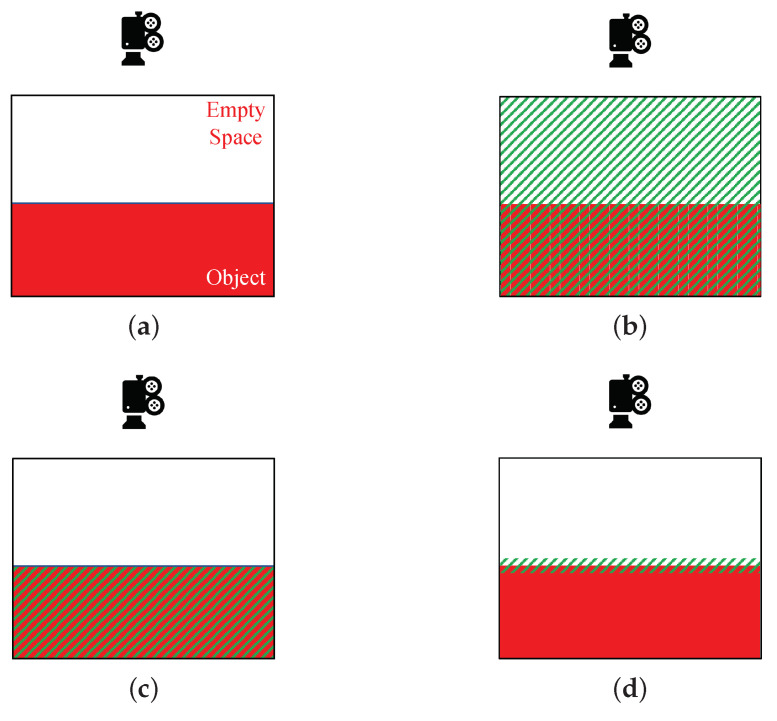
The focus regions of the loss functions (green masks for the focus regions). (**a**) The illustration of the example scene, where one object exists. (**b**) The scene loss. (**c**) The object loss. (**d**) The surface loss.

**Denoising Loss.** The Denoising Module is also trained in a supervised manner, considering the fusion accuracy on the whole scene, objects, and surface regions. The loss function is defined as follows:

(11) $$\begin{array}{c} L_{D}=\lambda_{1}^{D}L_{SPACE}+\lambda_{2}^{D}L_{OBJECT}+\lambda_{3}^{D}L_{SURFACE}, \end{array}$$

where $L_{SPACE}$, $L_{OBJECT}$, and $L_{SURFACE}$ are, respectively, for the losses of the whole scene, objects, and the surface regions (as shown in Figure 4). $\lambda_{1}^{D}$, $\lambda_{2}^{D}$, and $\lambda_{3}^{D}$ are the weights to adjust their relative importance.

$L_{SPACE}$ is defined as

(12) $$\begin{array}{c} L_{SPACE}=L_{1}\left(V,V^{\prime }\right), \end{array}$$

where *V* is the predicted scene volume while $V^{\prime }$ is the ground-truth volume.

Let $V_{OBJECT}\subseteq V$, and for each $v_{m}\in V_{OBJECT}$, $v_{m}^{\prime }\leq 0$, then

(13) $$\begin{array}{c} L_{OBJECT}=L_{1}\left(V_{OBJECT},V_{OBJECT}^{\prime }\right) \end{array}$$

Similarly, let $V_{SURFACE}\subseteq V$, and for each $v_{m}$ in $V_{SURFACE}$, $-S\leq v_{m}^{\prime }\leq S$, where *S* is a threshold of the surface range (we set *S* to 0.02), then

(14) $$\begin{array}{c} L_{SURFACE}=L_{1}\left(V_{SURFACE},V_{SURFACE}^{\prime }\right) \end{array}$$

We set the values of hyperparameter $\lambda_{1}^{D}$, $\lambda_{2}^{D}$, and $\lambda_{3}^{D}$ to 0.5, 0.25, and 0.25, respectively. The effects of object loss and surface loss are explored in the ablation study.

## 4. Experiments

In this section, we first explain the details of the experimental setup. Then we introduce the adopted datasets, with which both quantitative and qualitative results prove that our proposed method outperforms existing methods.

### 4.1. Experimental Setup

All the network models are implemented in PyTorch and trained with NVIDIA P100 GPU. The RMSprop optimization algorithm [31] is adopted with an initial learning rate of $10^{-4}$ and the momentum of 0.9, for both the fusion network and denoising network. The networks are trained sequentially, that is, the fusion network is pre-trained before the training of the denoising network. 10K frames sampled from ShapeNet dataset [32] are utilized for training the network.

### 4.2. Dataset and Noise Simulation

**ShapeNet** dataset [32] includes a large scale of synthetic 3D shapes, such as the plane, sofa and car. The ground-truth data, including depth maps, camera intrinsics and camera poses, can be obtained from the 3D shapes. Similar to RoutedFusion [4], we use the ShapeNet dataset to train the networks. To simulate the realistic noisy situation, not only depth maps but also camera poses are added random noises in the training process.

**CoRBS** dataset [33], a comprehensive RGB-D benchmark for SLAM, provides (i) real depth data and (ii) real color data, which are captured with a Kinect v2, (iii) a ground-truth trajectory of the camera that is obtained with an external motion capture system, and (iv) a ground-truth 3D model of the scene that is generated via an external 3D scanner. Totally, the dataset involves 20 image sequences of 4 different scenes.

**Noise Simulation**. As introduced in Section 3.2, we need the $\mu_{t}$, $\sigma_{t}$, $\mu_{r}$, and $\sigma_{r}$ parameters to mimic the real sensor noises. Since the CoRBS dataset provides not only real-scene data but also the ground-truth data, we adopt it to obtain the realistic pose noise for simulation. In order to measure the pose noise, we follow the calculation process of the commonly-used relative pose error (RPE) [34]. RPE is defined as the drift of the trajectory over a fixed time interval Δ. For a sequence of *n* frames, firstly, the relative pose error at time step *i* is calculated as follows:

(15) $$E_{i}={\left(I_{i}^{-1}I_{i+\Delta }\right)}^{-1}\left(J_{i}^{-1}J_{i+\Delta }\right),$$

where *I* is the ground-truth trajectory and *J* is the estimated trajectory. Then m=n−Δ individual relative pose error matrices can be obtained along the sequence. Generally, the RPE is considered as two components, i.e., RPE for translation matrix ($T=trans(E_{i})$) and RPE for rotation matrix ($R=rot(E_{i})$). We use the following formulas for obtaining the μ and σ parameters for the normal distribution.

(16) $$\mu_{t}=\frac{1}{m}\sum_{i=1}^{m}\left\|trans\left(E_{i}\right)\right\|$$

(17) $$\sigma_{t}=\sqrt{\frac{1}{m}\sum_{i=1}^{m}(\|trans\left(E_{i}\right){\left\|-\mu_{t}\right)}^{2}}$$

(18) $$\mu_{r}=\frac{1}{m}\sum_{i=1}^{m}∠rot\left(E_{i}\right)$$

(19) $$\sigma_{r}=\sqrt{\frac{1}{m}\sum_{i=1}^{m}{\left(∠rot\left(E_{i}\right)-\mu_{r}\right)}^{2}},$$

where $∠rot\left(E_{i}\right)=arccos\left(\frac{Tr(R)-1}{2}\right)$ and Tr(R) represents the sum of the diagonal elements of the rotation matrix *R*.

For the translation error, $\mu_{t}$ is 0.006 and $\sigma_{t}$ is 0.004, while for the rotation error, $\mu_{r}$ is 0.094 and $\sigma_{r}$ is 0.068, which are used in the noise simulation for our experiments. These parameters are also preferable in the training of DFusion model for actual uses, while they can also be increased a bit (better keeping $\mu_{t}$ and $\sigma_{t}$ no larger than 0.02, $\mu_{r}$ and $\sigma_{r}$ no larger than 0.2, with which the DFusion model can give good fusion results) if strong sensor noises are expected.

### 4.3. Evaluation Results

**Figure 5:**
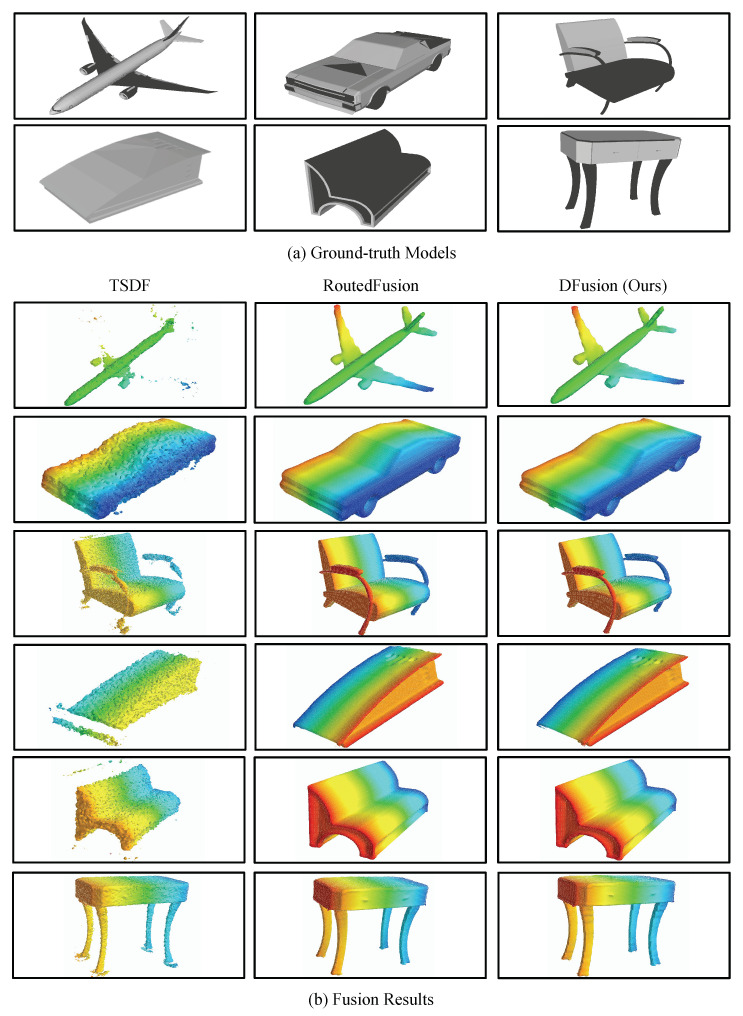
Fusion results on the ShapeNet dataset with depth noise added.

**Figure 6:**
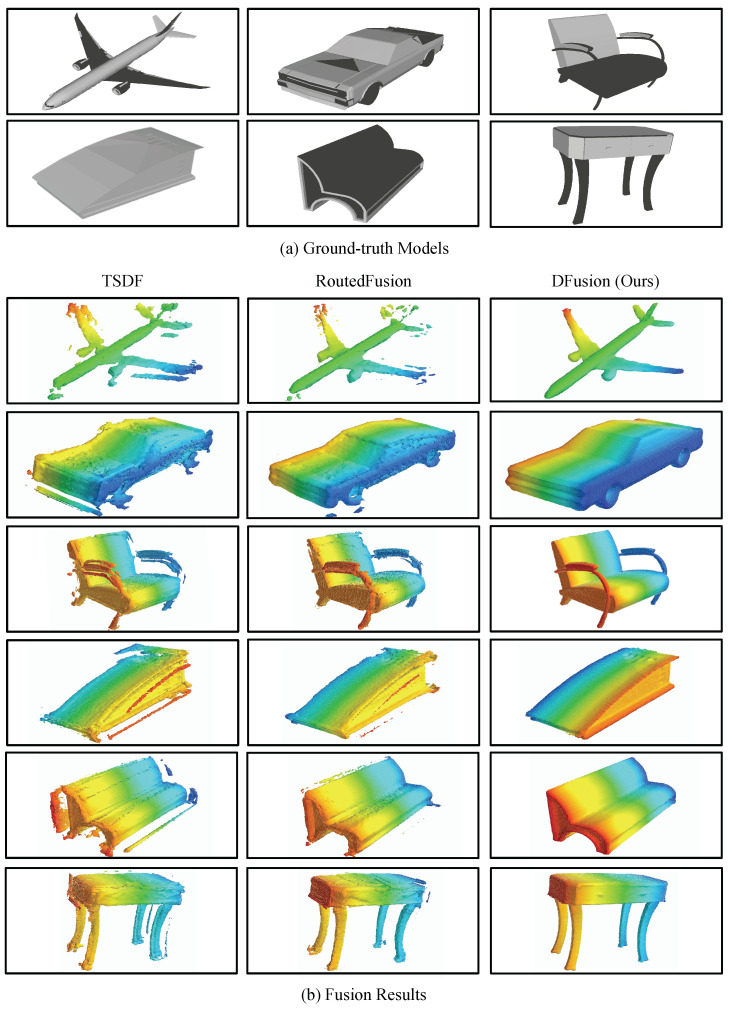
Fusion results on the ShapeNet dataset with pose noise added.

The experiments are conducted on ShapeNet and CoRBS datasets. For ShapeNet dataset, which involves the synthetic data, we add only depth noises and both depth noises and pose noises, respectively. The results are shown in Table 1 and Table 2. To compare with state-of-the-art methods, our method is evaluated with four metrics, i.e., the mean squared error (MSE), the mean absolute distance (MAD), intersection over union (IoU) and accuracy (ACC). MSE and MAD mainly focus on the distance between the estimated TSDF and the ground truth, while IoU and ACC quantify the occupancy of the estimation. According to the results, our method outperforms the state-of-the-art methods on all metrics for both scenarios. Especially when there exist both depth noises and pose noises, our method shows a significant advantage over other methods. When only depth noises exist, the RoutedFusion method and the proposed DFusion method have similar performance, while the latter shows a slight advantage due to the post-processing of the Denoising Module. Figure 5 and Figure 6 illustrate the fusion results on the ShapeNet dataset with depth noises or pose noises, respectively, which is more intuitive to show the advantages of DFusion method. Consistent with the metric results, we can see that DFusion can give clean and precise fusion for all these objects. Due to the use of deep learning models, RoutedFusion and DFusion both have satisfactory outputs when depth noises are added, as shown in Figure 5. However, when pose noises exist (as shown in Figure 6), the fusion results of RoutedFusion deteriorate a lot, while our DFusion model can still have a precise output.

**Figure 7:**
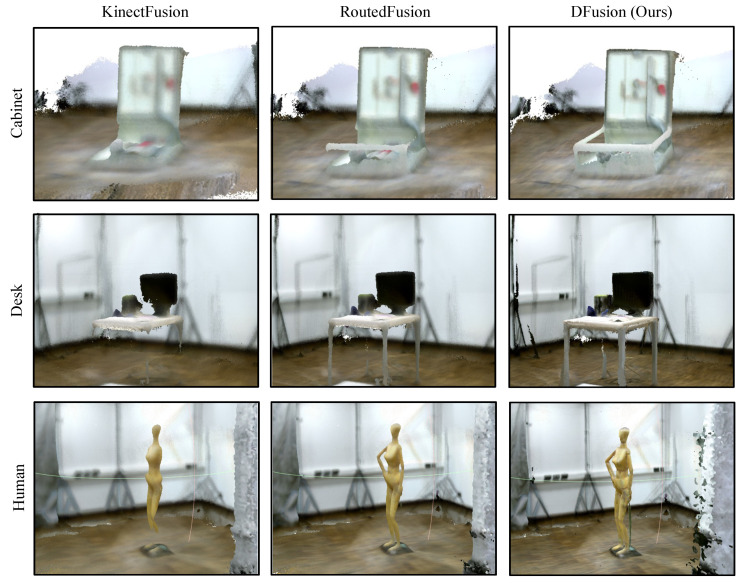
Fusion results on the CoRBS dataset. ICP algorithm [36] is used to obtain the sensor trajectory for RoutedFusion and DFusion.

For the CoRBS dataset, we choose four real scenes to perform the comparison with KinectFusion and RoutedFusion method. However, the pose information needs to be calculated before fusing the depth maps. KinectFusion method involves the process of calculating the pose information, which is the iterative closest point (ICP) algorithm [36]. Hence, to generate the TSDF volume, we use the ICP algorithm to obtain pose information for RoutedFusion and DFusion method, then compare the results on the MAD metric. The results are shown in Table 3. For all the scenes, our method achieves the best result. We also show some visualization results in Figure 7, which proves that our method can denoise the TSDF volume effectively and obtain more complete and smooth object models (note the cabinet edges, desk legs, and the human model arms).

### 4.4. Ablation Study

To verify the effectiveness of the proposed loss function, we perform an ablation study, which compares the results with other three variants of the loss function, i.e., the loss function without object loss, the loss function without surface loss and the loss function without both object and surface loss. The original loss is our default setting which involves space loss, object loss and surface loss. For all variants, the experiment is conducted on the ShapeNet dataset with both depth noises and pose noises added. The results are shown in Table 4. It can be seen that the original setting can achieve the best performance for all metrics, which demonstrates the effectiveness of the proposed loss functions.

## 5. Conclusions

In this paper, we propose a new depth fusion network, considering not only depth noises but also pose noises of depth sensors, which is more realistic in 3D reconstruction. To improve the fusion quality, a new CNN model is proposed after fusing the depth maps. A synthetic dataset and a real-scene dataset are adopted to verify the effectiveness of our method. It has been proved that our method outperforms existing depth fusion methods for both quantitative results and qualitative results.

One limitation of our proposed method is that it can only be used after all depth sequences have been obtained. Therefore, it cannot be deployed in systems that require real-time fusion. A possible solution is to involve incomplete depth sequences in the training process, where we may need to redesign the noise generation and model optimization methods, which can be one of the future objectives. In addition, DFusion may have some performance issues if it is only trained on a small dataset, as the Denoising Module requires enough training samples. Therefore, more works are needed to lower its data requirements.

## Figures and Tables

**Table 1:** Comparison results on ShapeNet (with only depth noise).

Methods	MSE	MAD	ACC	IoU
DeepSDF [35]	412.0	0.049	68.11	0.541
OccupacyNetworks [23]	47.5	0.016	86.38	0.509
TSDF Fusion [1]	10.9	0.008	88.07	0.659
RoutedFusion [4]	5.4	0.005	95.29	0.816
DFusion (Ours)	**3.5**	**0.003**	**96.12**	**0.847**

**Table 2:** Comparison results on ShapeNet (with depth noise and pose noise).

Methods	MSE	MAD	ACC	IoU
DeepSDF [35]	420.3	0.052	66.90	0.476
OccupacyNetworks [23]	108.6	0.037	77.34	0.453
TSDF Fusion [1]	43.4	0.020	80.45	0.582
RoutedFusion [4]	20.8	0.017	88.19	0.729
DFusion (Ours)	**6.1**	**0.006**	**95.08**	**0.801**

**Table 3:** Quantitative results (MAD) on the CoRBS dataset.

Methods	Human	Desk	Cabinet	Car
KinectFusion [5]	0.015	0.005	0.009	0.009
ICP + RoutedFusion [4]	0.014	0.005	0.008	0.009
ICP + DFusion (Ours)	**0.012**	**0.004**	**0.006**	**0.007**

**Table 4:** Variants of the proposed method (with depth noise and pose noise).

Methods	MSE	MAD	ACC	IoU
Without object loss	8.3	0.007	92.11	0.744
Without surface loss	7.5	**0.006**	91.83	0.769
Without object&surface loss	16.3	0.015	90.87	0.740
Original	**6.1**	**0.006**	**95.08**	**0.801**

## Data Availability

The data is from the public datasets.
